# Supplementary material for: A Global Screen for Assembly State Changes of the Mitotic Proteome by SEC-SWATH-MS
Source: Cell Syst. 2020 Feb 26;10(2):133–155.e6. doi: 10.1016/j.cels.2020.01.001 (PMC7042714; doi:10.1016/j.cels.2020.01.001)

P30101 | PDIA3_HUMAN | PDIA3 ERP57 ERP60 GRP58
Monomer MW [kDa]: 56.782 Monomer expected elution fraction: 45

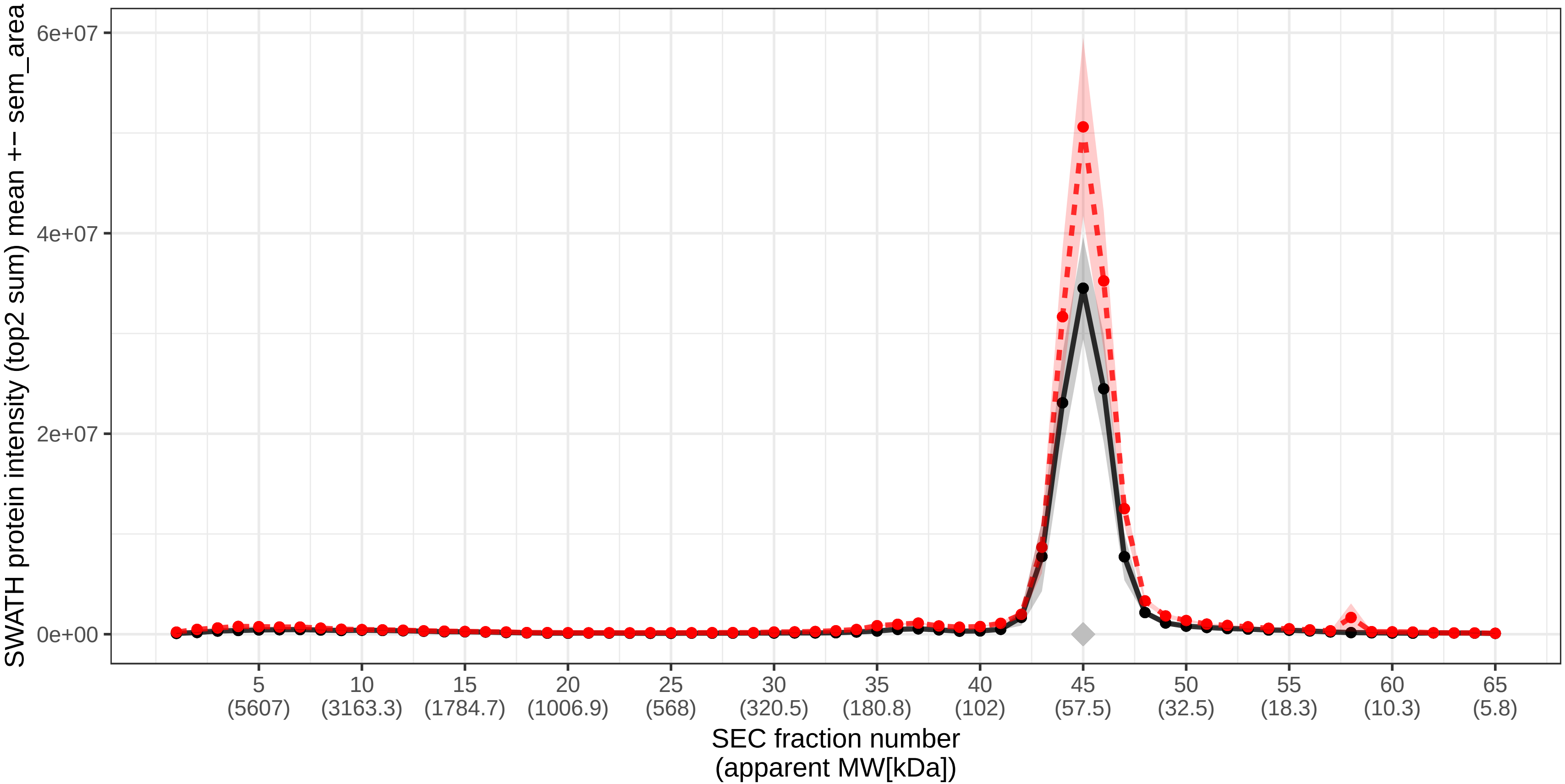

Supplement: Data S1. SEC-SWATH-MS Protein Chromatograms, Related to Figure 1 [file mmc6.zip › SECchrom_P30101_PDIA3_HUMAN_PDIA3_ERP57_ERP60_GRP58.pdf]
